# Supplementary material for: Impact of variation in functions and delivery on the effectiveness of behavioural and mood management interventions for smoking cessation in people with depression: protocol for a systematic review and meta-analysis
Source: BMJ Open. 2017 Nov 16;7(11):e018617. doi: 10.1136/bmjopen-2017-018617 (PMC5702022; doi:10.1136/bmjopen-2017-018617)
Supplement: Author's manuscript [file bmjopen-2017-018617.draft_revisions.pdf]

BMJ Open is committed to open peer review. As part of this commitment we make the peer review history of every article we publish publicly available.

When an article is published we post the peer reviewers' comments and the authors' responses online. We also post the versions of the paper that were used during peer review. These are the versions that the peer review comments apply to.

The versions of the paper that follow are the versions that were submitted during the peer review process. They are not the versions of record or the final published versions. They should not be cited or distributed as the published version of this manuscript.

BMJ Open is an open access journal and the full, final, typeset and author-corrected version of record of the manuscript is available on our site with no access controls, subscription charges or pay-per-view fees (http://bmjopen.bmj.com).

If you have any questions on BMJ Open's open peer review process please email editorial.bmjopen@bmj.com

BMJ Open

# Impact of variation in intervention delivery and functions on the effectiveness of behavioural and mood management smoking cessation interventions for smokers with depression: A systematic review and meta-analysis protocol.

For peer review only



| | |
|---|---|
| Journal: | *BMJ Open* |
| Manuscript ID | Draft |
| Article Type: | Protocol |
| Date Submitted by the Author: | n/a |





| | |
|---|---|
| Complete List of Authors: | Taylor, Gemma; University of Bristol, Medical Research Council Integrative Epidemiology Unit, UK Centre for Tobacco and Alcohol Studies, School of Experimental Psychology<br>Aveyard, Paul; University of Oxford, UK Centre for Tobacco and Alcohol Studies, Primary Care Health Sciences<br>Van der Meer, Regina ; Public Health Service of Haaglanden (GGD Haaglanden), Epidemiology; Maastricht University, CAPHRI<br>Toze, Daniel; University of Bristol, Medical Research Council Integrative Epidemiology Unit, UK Centre for Tobacco and Alcohol Studies, School of Experimental Psychology<br>Stuijfzand, Bobby ; University of Bristol, Jean Golding Institute for Data-Intensive Research<br>Kessler, David; University of Bristol, Centre for Academic Mental Health<br>Munafo, Marcus; University of Bristol, Medical Research Council Integrative Epidemiology Unit, UK Centre for Tobacco and Alcohol Studies, School of Experimental Psychology |





| | |
|---|---|
| <b>Primary Subject Heading</b>: | Addiction |
| Secondary Subject Heading: | Mental health |
| Keywords: | Tobacco, Smoking cessation, Depression, Systematic review, Protocol, Intervention |
| | |



SCHOLARONE™
Manuscripts

## TITLE

Impact of variation in intervention delivery and functions on the effectiveness of behavioural and mood management smoking cessation interventions for smokers with depression: A systematic review and meta-analysis protocol.


## AUTHORS

Gemma Taylor, Research Fellow [1,2]; Paul Aveyard, Professor of Behavioural Medicine [3]; Regina Van der Meer, Epidemiologist, Health Scientist [4,5]; Daniel Toze, Research Assistant [1,2]; Bobby Stuijfzand, Data Science Specialist [6]; David Kessler, Reader in Primary Care [7]; Marcus Munafò, Professor of Biological Psychology [1,2]

## AFFILIATIONS

[1] Medical Research Council Integrative Epidemiology Unit at the University of Bristol, Bristol, BS8 2BN, United Kingdom.

[2] UK Centre for Tobacco and Alcohol Studies, School of Experimental Psychology, University of Bristol, 12a Priory Road, Bristol, BS8 1TU, United Kingdom.

[3] UK Centre for Tobacco and Alcohol Studies, Nuffield Department of Primary Care Health Sciences, University of Oxford, Oxford, OX1 2ET, United Kingdom.

[4] Public Health Service of Haaglanden (GGD Haaglanden), Epidemiology, The Hague, Netherlands.

[5] Maastricht University, CAPHRI, Maastricht, Netherlands

[6] Jean Golding Institute for Data-Intensive Research, University of Bristol, Royal Fort House, Bristol, BS8 1UH, United Kingdom.

[7] Centre for Academic Mental Health, University of Bristol, Oakfield House, Oakfield Grove, Bristol, BS8 2BN, United Kingdom.

## CORRESPONDING AUTHOR

Gemma Taylor, gemma.taylor@bristol.ac.uk, MRC Integrative Epidemiology Unit, UK Centre for Tobacco and Alcohol Studies, School of Experimental Psychology, University of Bristol, 12a Priory Road, Bristol, BS8 1TU, United Kingdom.



## WORD COUNT

1379

## KEYWORDS

Tobacco, Smoking cessation, Depression, Systematic review, Protocol, Intervention


For peer review only

**ABSTRACT**

**Introduction**

Tobacco is the world's leading preventable cause of disease and death. People with depression are twice as likely to smoke and are less responsive to standard tobacco treatments as compared to the general population. A Cochrane systematic review (Van der Meer 2013) of randomised controlled trials of smoking cessation treatment for smokers with current or historical depression found that adding psychosocial mood management to usual smoking treatment improved quit rates. However, the review did not examine if variation in intervention delivery or intervention functions impacted on treatment effectiveness.

With the aim of providing information to develop tailored approaches to treating smoking for people with depression we will add-on to the Cochrane review in three ways: 1) Use the Template for Intervention Description and Replication checklist (TIDieR) to determine if variations in delivery of mood management components impact on intervention effectiveness, 2) Use the Taxonomy of Behaviour Change techniques for smoking cessation to examine which behaviour change functions are most effective for smoking cessation in people with current depression, 3) Examine the difference in change in depression scores between intervention and control arms.

**Methods and Analysis**

This review has been registered on PROSPERO (Review No. 70741). We will include randomised controlled trials of smokers with current depression as identified by the Cochrane review (Van der Meer 2013) and the in-progress update of the Cochrane review. We will use meta-regression to examine the impact of intervention components and behaviour change functions on treatment effectiveness, and a meta-analysis of the difference in change in depression scores between treatment arms from baseline to follow-up.

**Ethics and Dissemination**

Ethical approval is not required for this study. We will disseminate the findings of this work at international and national conferences, and to the UK Centre for Tobacco and Alcohol Studies Smokers' Panel.

For peer review only

STRENGTHS AND LIMITATIONS OF THIS STUDY

- We will examine the impact of variation in intervention delivery and functions on treatment effectiveness using peer-reviewed checklists: The Behaviour Change Taxonomy (Michie et al, 2011), and Template for Intervention Description and Replication (TIDieR, Hoffmann et al, 2014).
- If analyses are possible, this study will provide causal effects of smoking treatment on depression symptoms in people with depression.
- May suffer from low power.
- May suffer from publication bias.

For peer review only

## BACKGROUND

Tobacco is the world's leading preventable cause of disease and death.[1] In the UK and in other developed nations smoking prevalence has declined substantially in the general population, but has remained largely unchanged in those with mental health problems resulting in an excess burden of smoking-related mortality in this group.[2,3] People with depression are twice as likely to smoke[4,5] and are less responsive to standard tobacco treatments than are the general population[6,7] leading to urgent calls for targeted smoking interventions.[8]

The Cochrane Group conducted a systematic review and meta-analysis of smoking cessation interventions for smokers with past or present depression. The review included pharmacological and behavioural interventions to aid cessation and found that adding psychosocial mood management to a usual smoking treatment (e.g., nicotine replacement therapy, telephone counselling, self-help website) moderately increased smoking cessation rates in people with current depression compared to usual smoking treatment alone, reporting a risk ratio of 1.47 (95% confidence interval: 1.13 to 1.92).[9] The review highlighted the importance of adding psychosocial techniques to handle depressive symptoms in standard smoking treatments for people with depression. However, in the meta-analysis there was variation between the included studies' direction of effect and it is possible that this variation may be in part related to differences in intervention delivery, intervention functions, or tailoring, for example. Further investigation into these potential modifiers will provide useful information for development of smoking cessation interventions for people with current depression.[9]

In addition, the review did not examine the impact of behavioural or psychological smoking cessation interventions on depression symptoms. This is an important question as many clinicians believe that smoking may offer mental health benefits, or that their patients' mental health may deteriorate upon cessation.[10] However, there are data from meta-analyses of cohort studies indicating that quitting smoking may improve depression,[11] but due to common pitfalls of observational cohort studies one cannot be sure that this is a causal association. If treating smoking is found to not worsen depression, then these data can be used to assure clinicians that they are not causing psychological harm by helping their patients to quit smoking.

In our review, we aim to add-on to the previous Cochrane review in three ways. We will:

1) Use the Template for Intervention Description and Replication checklist (TIDieR)[12] to determine if variations in delivery of mood management components impact on intervention effectiveness.
2) Use the Taxonomy of Behaviour Change techniques for smoking cessation[13] to examine which behaviour change functions are most effective for smoking cessation in people with current depression.
3) Examine the difference in change in depression scores between intervention and control arms.

For peer review only

## METHODS

The study protocol will be registered in advance on the International Prospective Register of Systematic Reviews (PROSPERO; http://www.crd.york.ac.uk/PROSPERO/), uploaded to bioRxiv (http://biorxiv.org/), and submitted for publication to a peer-reviewed journal. All methods and study reporting will adhere to guidance described within the Cochrane Handbook for Systematic Reviews and Meta-analyses of Randomised Controlled Trials.[14]

**Inclusion criteria**

- Study design: Randomised controlled trials only;
- Participants: Daily smokers with current depression, any definition of depression, no restrictions by physical or mental comorbidities;
- Intervention: Any smoking cessation intervention;
- Intervention delivery: Self-help, individual, group, internet;
- Control: Any (e.g., including self-help, no treatment, etc.);
- Outcome: Any ascertainment of smoking cessation;
- Follow-up: Follow up at a minimum of 6- months from the quit date.

**Outcomes:**

- Smoking status at final follow-up;
- Change in depression scores from baseline to final follow-up.

**Search strategy**

We will include relevant studies identified by a previously conducted Cochrane review of smoking cessation interventions for people with depression.[9] This review will be updated this year and we will also include relevant studies from the updated version of the review.

**Data extraction**

We will extract the following data from included trials:

Trial methods:

Study design, setting, country, randomisation methods.

Participants:

Number of participants per intervention group, definition of depression, type of smoker, comorbid conditions, age, sex, ethnicity, level of education, nicotine dependence, mean/median number of cigarettes per day (CPD), depression type and severity.

Interventions:

Description of the interventions, the number of and function of behaviour change techniques used (where sufficient details are not reported in text, we will attempt to obtain intervention protocols), presence or absence of TIDieR checklist items.

For peer review only

Control:

Description of the control, the number of and function of behaviour change techniques used (where sufficient details are not reported in text, we will attempt to obtain intervention protocols), presence or absence of TIDieR checklist items.

Outcomes:

Smoking cessation status, biochemical validation, depression scores, length of follow-up.

**Coding of Template for Intervention Description and Replication checklist (TIDieR)**

For study aim 1 we will use the Template for Intervention Description and Replication checklist (TIDieR)[12] to determine if variations in delivery of mood management components impact on intervention effectiveness. We will use a modified version of the template as not all questions on the checklist are useful in the context of this study (e.g. "Describe any rationale, theory, or goal of the elements essential to the intervention"). Coding will be conducted separately by two researchers to confirm agreement.

**Coding of behaviour change intervention functions:**

We will categorise behaviour change techniques according to their function and record whether the function was either absent or present during intervention delivery[13] (Table 2). Coding will be conducted separately by two researchers to confirm agreement.

**Measures of treatment effect**

Smoking cessation (Study aims 1 & 2):

We will present treatment effects as risk ratios (RR) and 95% confidence intervals[14]. RRs will be calculated as follows: (number of participants who quit smoking in the intervention group/number of participants randomised to intervention group) divided by (number of participants who quit smoking in control group/number of participants randomised to the control group).

Difference in change in depression scores between trial arms (Study aim 3):

We will present the standardised mean difference (SMD), and 95% confidence intervals of change in depression scores between treatment arms, from baseline to follow-up.

**Analysis**

All analyses will be conducted using Stata 14.

Study aim 1 - Do variations in delivery of mood management components impact on intervention effectiveness:

If there are sufficient data, we will conduct random effects meta-regression using the `metareg` command[15] in which modified TIDieR Checklist items (see Table 1) will be regressed on the study's effect estimate. First, univariate analyses will be conducted to determine the association between each item and the study effect size. Subsequently, items with the strongest association will be added to the meta-regression model first, and all other variables will be added in turn regardless of significance in the univariate model.

For peer review only

Study aim 2 - Use the Taxonomy of Behaviour Change techniques for smoking cessation to examine which behaviour change functions are most effective for smoking cessation in people with current depression:

If there are sufficient data, we will conduct random effects meta-regression using the `metareg` command[15] in which behaviour change functions (see Table 2) will be regressed on the study's effect estimate. First, univariate analyses will be conducted to determine the association between each intervention component and the study effect size. Subsequently, variables with the strongest association will be added to the meta-regression model first, and all other variables will be added in turn regardless of significance in the univariate model.

Study aim 3 - Examine the difference in change in depression scores between intervention and control arms:

If there are sufficient data, we will use a generic inverse variance random effects model to pool the standardised mean difference (SMD) of change in depression scores in treatment and control arms, from baseline to follow-up. We will use a random effects model as it incorporates heterogeneity both within and between studies.

Statistical heterogeneity:

We will quantify statistical heterogeneity using $I^2$ which describes the percentage (%) of between-study variability due to heterogeneity rather than chance; values over 50% suggest substantial heterogeneity, and values over 75% suggest considerable heterogeneity.[14] $Tau^2$ will be used to test whether differences between studies' effect estimates are compatible with chance alone.[16]

Sensitivity and subgroup analyses:

We will conduct sensitivity analyses to examine if the following study characteristics influence the meta-analysis results: study quality (as measured by Cochrane's Risk of Bias tool), loss-to-follow-up, and severity of depression.

Assessment of publication bias:

We will examine funnel plots for evidence of asymmetry and conduct egger tests for evidence of small study bias using the `metabias` command.[15]

For peer review only

**ETHICAL APPROVAL**

Ethical approval is not required for this study.

**DISSEMINATION**

We will disseminate the findings of this work at international and national conferences, and to the UK Centre for Tobacco and Alcohol Studies Smokers' Panel.

**ACKNOWLEDGMENTS**

None.

**COMPETING INTERESTS**

All authors completed ICMJE Form for Disclosure of Potential Conflicts of Interest. GT, DT, RV, DK, BS report no competing interests. PA reports non-financial support from GSK, outside the submitted work. MM reports grants from Pfizer, outside the submitted work.

**FUNDING**

GT is funded by Cancer Research UK Population Researcher Postdoctoral Fellowship award (reference: C56067/A21330). PA is supported by the NIHR Biomedical Research Centre and the NIHR CLAHRC. RV reports no funding for this work. BS and DK are funded by The University of Bristol. MRM would like to acknowledge funding from The MRC Integrative Epidemiology Unit at the University of Bristol which is supported by the Medical Research Council and the University of Bristol (MC_UU_12013/6). GT, MRM and PA are members of the UK Centre for Tobacco and Alcohol Studies, a UKCRC Public Health Research: Centre of Excellence. Funding from the British Heart Foundation, Cancer Research UK, Economic and Social Research Council, Medical Research Council, and the National Institute for Health Research, under the auspices of the UK Clinical Research Collaboration, is gratefully acknowledged. The funders have had no role in developing the protocol or study design.

**CONTRIBUTORSHIP STATEMENT**

All authors contributed to writing the manuscript and reviewed the final draft. GT, PA, RV, DK, BS, MM all contributed towards study design. DT is contributing to data extraction. GT and MM act as the guarantors of this review

For peer review only

## REFERENCES

1. Organization WH, Press WHO. *WHO Report on the Global Tobacco Epidemic: Warning about the Dangers of Tobacco*. Geneva, Switzerland; 2011.
2. Physicians RC of, Psychiatrists RC of, Physicians RC of. *Smoking and Mental Health*. London, UK; 2013.
3. Chang C, Hayes R, Perera G, et al. Life expectancy at birth for people with serious mental illness and other major disorders from a secondary mental health care case register in London. *PLoS One*. 2011;6(5):e19590. doi:doi:10.1371/journal.pone.0019590.
4. Lawrence D, Mitrou F, Zubrick SR, et al. Smoking and mental illness: results from population surveys in Australia and the United States. *BMC Public Health*. 2009;9(1):285. doi:10.1186/1471-2458-9-285.
5. Lasser K, Boyd J, Woolhandler S, Himmelstein D, McCormick D, Bor D. Smoking and mental illness: a population-based prevalence study. *JAMA*. 2000;284(20):2606-2610.
6. Johnson KA, Bonn-Miller MO, Leyro TM, Zvolensky MJ. Anxious Arousal and Anhedonic Depression Symptoms and the Frequency of Current Marijuana Use: Testing the Mediating Role of Marijuana-Use Coping Motives Among Active Users. *J Stud Alcohol Drugs*. 2009;70(4):543-550. wos:000267230100007.
7. Zhou X, Nonnemaker J, Sherrill B, Gilsenan AW, Coste F, West R. Attempts to quit smoking and relapse: factors associated with success or failure from the ATTEMPT cohort study. *Addict Behav*. 2009;34(4):365-373. http://ukpmc.ac.uk/abstract/MED/19097706.
8. Social Care LG and CPD. *Closing the Gap: Priorities for Essential Change in Mental Health* . London, UK: Department of Health; 2014.
9. van der Meer RM, Willemsen MC, Smit F, Cuijpers P. Smoking cessation interventions for smokers with current or past depression. *Cochrane Libr*. 2013.
10. Sheals K, Tombor I, McNeill A, Shahab L. A mixed-method systematic review and meta-analysis of mental health professionals' attitudes toward smoking and smoking cessation amongst people with mental illnesses. *Addiction*. 2016:n/a-n/a. doi:10.1111/add.13387.
11. Taylor G, McNeill A, Girling A, Farley A, Lindson N, Aveyard P. Change in mental health after smoking cessation: systematic review and meta-analysis. *BMJ*. 2014;348.
12. Hoffmann TC, Glasziou PP, Boutron I, et al. Better reporting of interventions: template for intervention description and replication (TIDieR) checklist and guide. *BMJ*. 2014;348:g1687. http://www.ncbi.nlm.nih.gov/pubmed/24609605. Accessed May 30, 2017.
13. Michie S, Hyder N, Walia A, West R. Development of a taxonomy of behaviour change techniques used in individual behavioural support for smoking cessation. *Addict Behav*. 2011;36:315-319.
14. Higgins J, Green S. *Cochrane Handbook for Systematic Reviews of Interventions Version 5.1.0*. The Cochrane Collaboration; 2011.
15. Sterne, Jonathan AC, Michael J. Bradburn and ME. Meta–Analysis in Stata™. In: *Systematic Reviews in Health Care: Meta-Analysis in Context*. Second Edi. ; 2008:347-369.
16. Harbord R, Higgins J. METAREG: Stata module to perform meta-analysis regression. *Stat Softw Components*. 2009. http://ideas.repec.org/c/boc/bocode/s446201.html%5Cnhttp://fmwww.bc.edu/repec/bocode/m/metareg.ado%5Cnhttps://ideas.repec.org/c/boc/bocode/s446201.html.

For peer review only

**TABLES**

| Table 1. Modified version of Template for Intervention Description and Replication checklist (TIDieR)[12] for use in meta-regression analysis* | |
|---|---|
| **Item** | **Categories** |
| Materials for mood management (i.e., physical or informational materials used) | Paper-based information, website, homework, diary, audio information, etc. |
| Procedures for mood management: activities, procedures or activities used in the intervention to support activities | Relaxation techniques, mood monitoring, etc. |
| Did the participant see the same intervention provider for all mood management sessions? | Yes, no |
| Mood management provider | Nurse, psychologist, GP, counsellor, etc. |
| Training given to intervention provider? | Yes, no |
| Level of education of intervention provider | BSc, MSc, PhD |
| Mode of mood management intervention delivery | Individual, group |
| Location of mood management intervention | Hospital, participant's home, GP surgery, university, etc. |
| Number of mood management sessions | Continuous variable |
| Length of mood management session (minutes) | Continuous variable |
| Was the mood management intervention tailored to participant? | Yes, no |
| Number of mood management sessions tailored to participant? | Continuous variable |
| Was participant adherence to mood management intervention measured? | Yes, no |
| Did participants to adhere to mood management intervention. | Yes, no, or % |
| Was therapist adherence to mood management intervention measured? (Y/N) | Yes, no |
| Did therapists adhere to mood management programme? (Y/N) | Yes, no, or % |
| * The categories are likely to be further developed during data extraction to include new items. | |

For peer review only

| Table 2. Example of Behaviour change functions and techniques[13] | |
|---|---|
| **Behaviour change function** | **Examples of technique** |
| Specific focus on behaviour and addressing motivation | Provide information on consequences of smoking and smoking cessation |
| | Boost motivation and self-efficacy |
| | Provide feedback on current behaviour |
| Specific focus on behaviour and maximising self-regulatory capacity/skills | Advise on changing routine |
| | Advise on environmental restructuring |
| | Set graded tasks |

For peer review only

**PRISMA-P (Preferred Reporting Items for Systematic review and Meta-Analysis Protocols) 2015 checklist: recommended items to address in a systematic review protocol***

| Section and topic | Item No | Checklist item | Page |
|---|---|---|---|
| **ADMINISTRATIVE INFORMATION** | | | |
| Title: | | | |
| Identification | 1a | Identify the report as a protocol of a systematic review | 1 |
| Update | 1b | If the protocol is for an update of a previous systematic review, identify as such | n/a |
| Registration | 2 | If registered, provide the name of the registry (such as PROSPERO) and registration number | 2, 5 |
| Authors: | | | |
| Contact | 3a | Provide name, institutional affiliation, e-mail address of all protocol authors; provide physical mailing address of corresponding author | 1 |
| Contributions | 3b | Describe contributions of protocol authors and identify the guarantor of the review | 8 |
| Amendments | 4 | If the protocol represents an amendment of a previously completed or published protocol, identify as such and list changes; otherwise, state plan for documenting important protocol amendments | n/a |
| Support: | | | |
| Sources | 5a | Indicate sources of financial or other support for the review | 8 |
| Sponsor | 5b | Provide name for the review funder and/or sponsor | 8 |
| Role of sponsor or funder | 5c | Describe roles of funder(s), sponsor(s), and/or institution(s), if any, in developing the protocol | 8 |
| **INTRODUCTION** | | | |
| Rationale | 6 | Describe the rationale for the review in the context of what is already known | 4 |
| Objectives | 7 | Provide an explicit statement of the question(s) the review will address with reference to participants, interventions, comparators, and outcomes (PICO) | 4 |
| **METHODS** | | | |
| Eligibility criteria | 8 | Specify the study characteristics (such as PICO, study design, setting, time frame) and report characteristics (such as years considered, language, publication status) to be used as criteria for eligibility for the review | 5-6 |
| Information sources | 9 | Describe all intended information sources (such as electronic databases, contact with study authors, trial registers or other grey literature sources) with planned dates of coverage | 5 |
| Search strategy | 10 | Present draft of search strategy to be used for at least one electronic database, including planned limits, such that it could be repeated | n/a |

For peer review only

| | | | |
|---|---|---|---|
| Study records: | | | |
| Data management | 11a | Describe the mechanism(s) that will be used to manage records and data throughout the review | 6 |
| Selection process | 11b | State the process that will be used for selecting studies (such as two independent reviewers) through each phase of the review (that is, screening, eligibility and inclusion in meta-analysis) | n/a |
| Data collection process | 11c | Describe planned method of extracting data from reports (such as piloting forms, done independently, in duplicate), any processes for obtaining and confirming data from investigators | 6 |
| Data items | 12 | List and define all variables for which data will be sought (such as PICO items, funding sources), any pre-planned data assumptions and simplifications | 6 |
| Outcomes and prioritization | 13 | List and define all outcomes for which data will be sought, including prioritization of main and additional outcomes, with rationale | 5-6 |
| Risk of bias in individual studies | 14 | Describe anticipated methods for assessing risk of bias of individual studies, including whether this will be done at the outcome or study level, or both; state how this information will be used in data synthesis | 7 |
| Data synthesis | 15a | Describe criteria under which study data will be quantitatively synthesised | 6-7 |
| | 15b | If data are appropriate for quantitative synthesis, describe planned summary measures, methods of handling data and methods of combining data from studies, including any planned exploration of consistency (such as $I^2$, Kendall's τ) | 6-7 |
| | 15c | Describe any proposed additional analyses (such as sensitivity or subgroup analyses, meta-regression) | 7 |
| | 15d | If quantitative synthesis is not appropriate, describe the type of summary planned | 6-7 |
| Meta-bias(es) | 16 | Specify any planned assessment of meta-bias(es) (such as publication bias across studies, selective reporting within studies) | 6-7 |
| Confidence in cumulative evidence | 17 | Describe how the strength of the body of evidence will be assessed (such as GRADE) | n/a |

*** It is strongly recommended that this checklist be read in conjunction with the PRISMA-P Explanation and Elaboration (cite when available) for important clarification on the items. Amendments to a review protocol should be tracked and dated. The copyright for PRISMA-P (including checklist) is held by the PRISMA-P Group and is distributed under a Creative Commons Attribution Licence 4.0.**

*From: Shamseer L, Moher D, Clarke M, Ghersi D, Liberati A, Petticrew M, Shekelle P, Stewart L, PRISMA-P Group. Preferred reporting items for systematic review and meta-analysis protocols (PRISMA-P) 2015: elaboration and explanation. BMJ. 2015 Jan 2;349(jan02 1):g7647.*

For peer review only

BMJ Open

# Impact of variation in intervention delivery and functions on the effectiveness of behavioural and mood management smoking cessation interventions for smokers with depression: A systematic review and meta-analysis protocol.


| | |
|---|---|
| Journal: | *BMJ Open* |
| Manuscript ID | bmjopen-2017-018617.R1 |
| Article Type: | Protocol |
| Date Submitted by the Author: | 01-Sep-2017 |


| | |
|---|---|
| Complete List of Authors: | Taylor, Gemma; University of Bristol, Medical Research Council Integrative Epidemiology Unit, UK Centre for Tobacco and Alcohol Studies, School of Experimental Psychology<br>Aveyard, Paul; University of Oxford, UK Centre for Tobacco and Alcohol Studies, Primary Care Health Sciences<br>Van der Meer, Regina ; Public Health Service of Haaglanden (GGD Haaglanden), Epidemiology; Maastricht University, CAPHRI<br>Toze, Daniel; University of Bristol, Medical Research Council Integrative Epidemiology Unit, UK Centre for Tobacco and Alcohol Studies, School of Experimental Psychology<br>Stuijfzand, Bobby ; University of Bristol, Jean Golding Institute for Data-Intensive Research<br>Kessler, David; University of Bristol, Centre for Academic Mental Health<br>Munafo, Marcus; University of Bristol, Medical Research Council Integrative Epidemiology Unit, UK Centre for Tobacco and Alcohol Studies, School of Experimental Psychology |


| | |
|---|---|
| <b>Primary Subject Heading</b>: | Addiction |
| Secondary Subject Heading: | Mental health |
| Keywords: | Tobacco, Smoking cessation, Depression, Systematic review, Protocol, Intervention |


SCHOLARONE™
Manuscripts

**TITLE**

# Impact of variation in intervention delivery and functions on the effectiveness of behavioural and mood management smoking cessation interventions for smokers with depression: A systematic review and meta-analysis protocol.

**AUTHORS**

Gemma Taylor, Research Fellow [1,2]; Paul Aveyard, Professor of Behavioural Medicine [3]; Regina Van der Meer, Epidemiologist, Health Scientist [4,5]; Daniel Toze, Research Assistant [1,2]; Bobby Stuijfzand, Data Science Specialist [6]; David Kessler, Reader in Primary Care [7]; Marcus Munafò, Professor of Biological Psychology [1,2]

**AFFILIATIONS**

[1] Medical Research Council Integrative Epidemiology Unit at the University of Bristol, Bristol, BS8 2BN, United Kingdom.

[2] UK Centre for Tobacco and Alcohol Studies, School of Experimental Psychology, University of Bristol, 12a Priory Road, Bristol, BS8 1TU, United Kingdom.

[3] UK Centre for Tobacco and Alcohol Studies, Nuffield Department of Primary Care Health Sciences, University of Oxford, Oxford, OX1 2ET, United Kingdom.

[4] Public Health Service of Haaglanden (GGD Haaglanden), Epidemiology, The Hague, Netherlands.

[5] Maastricht University, CAPHRI, Maastricht, Netherlands

[6] Jean Golding Institute for Data-Intensive Research, University of Bristol, Royal Fort House, Bristol, BS8 1UH, United Kingdom.

[7] Centre for Academic Mental Health, University of Bristol, Oakfield House, Oakfield Grove, Bristol, BS8 2BN, United Kingdom.

**CORRESPONDING AUTHOR**

Gemma Taylor, gemma.taylor@bristol.ac.uk, MRC Integrative Epidemiology Unit, UK Centre for Tobacco and Alcohol Studies, School of Experimental Psychology, University of Bristol, 12a Priory Road, Bristol, BS8 1TU, United Kingdom.

**WORD COUNT**

1379

**KEYWORDS**

Tobacco, Smoking cessation, Depression, Systematic review, Protocol, Intervention

For peer review only

**ABSTRACT**

**Introduction**

Tobacco is the world's leading preventable cause of disease and death. People with depression are twice as likely to smoke and are less responsive to standard tobacco treatments as compared to the general population. A Cochrane systematic review (Van der Meer 2013) of randomised controlled trials of smoking cessation treatment for smokers with current or historical depression found that adding psychosocial mood management to usual smoking treatment improved quit rates. However, the review did not examine if variation in intervention delivery or intervention functions impacted on treatment effectiveness.

With the aim of providing information to develop tailored approaches to treating smoking for people with depression we will add-on to the Cochrane review in three ways: 1) Use the Template for Intervention Description and Replication checklist (TIDieR) to determine if variations in delivery of mood management components impact on intervention effectiveness, 2) Use the Taxonomy of Behaviour Change techniques for smoking cessation to examine which behaviour change functions are most effective for smoking cessation in people with current depression, 3) Examine the difference in change in depression scores between intervention and control arms.

**Methods and Analysis**

This review has been registered on PROSPERO (ID: CRD42017070741). We will include randomised controlled trials of smokers with current depression as identified by the Cochrane review (Van der Meer 2013) and the in-progress update of the Cochrane review. We will use meta-regression to examine the impact of intervention components and behaviour change functions on treatment effectiveness, and a meta-analysis of the difference in change in depression scores between treatment arms from baseline to follow-up.

**Ethics and Dissemination**

Ethical approval is not required for this study. We will disseminate the findings of this work at international and national conferences, and to the UK Centre for Tobacco and Alcohol Studies Smokers' Panel.

For peer review only

**STRENGTHS AND LIMITATIONS OF THIS STUDY**

- We will examine the impact of variation in intervention delivery and functions on treatment effectiveness using peer-reviewed checklists: The Behaviour Change Taxonomy (Michie et al, 2011), and Template for Intervention Description and Replication (TIDieR, Hoffmann et al, 2014).
- If analyses are possible, this study will provide causal effects of smoking treatment on depression symptoms in people with depression.
- May suffer from low power.
- May suffer from publication bias.

Not all intervention details may be reported. We will request intervention manuals from study authors, however these may not always be possible to obtain.

For peer review only

## BACKGROUND

Tobacco is the world's leading preventable cause of disease and death.[1] In the UK and in other developed nations smoking prevalence has declined substantially in the general population, but has remained largely unchanged in those with mental health problems resulting in an excess burden of smoking-related mortality in this group.[2,3] People with depression are twice as likely to smoke[4,5] and are less responsive to standard tobacco treatments than are the general population[6,7] leading to urgent calls for targeted smoking interventions.[8]

The Cochrane Group conducted a systematic review and meta-analysis of smoking cessation interventions for smokers with past or present depression. The review included pharmacological and behavioural interventions to aid cessation and found that adding psychosocial mood management to a usual smoking treatment (e.g., nicotine replacement therapy, telephone counselling, self-help website) moderately increased smoking cessation rates in people with current depression compared to usual smoking treatment alone, reporting a risk ratio of 1.47 (95% confidence interval: 1.13 to 1.92).[9] The review highlighted the importance of adding psychosocial techniques to handle depressive symptoms in standard smoking treatments for people with depression. However, in the meta-analysis there was variation between the included studies' direction of effect and it is possible that this variation may be in part related to differences in intervention delivery, intervention functions, or tailoring, for example. Further investigation into these potential modifiers will provide useful information for development of smoking cessation interventions for people with current depression.[9]

In addition, the review did not examine the impact of behavioural or psychological smoking cessation interventions on depression symptoms. This is an important question as many clinicians believe that smoking may offer mental health benefits, or that their patients' mental health may deteriorate upon cessation.[10] However, there are data from meta-analyses of cohort studies indicating that quitting smoking may improve depression,[11] but due to common pitfalls of observational cohort studies one cannot be sure that this is a causal association. If treating smoking is found to not worsen depression, then these data can be used to assure clinicians that they are not causing psychological harm by helping their patients to quit smoking.

In our review, we aim to add-on to the 2013 Cochrane review[9] in three ways. We will:

1) Use the Template for Intervention Description and Replication checklist (TIDieR)[12] to determine if variations in delivery of mood management components impact on intervention effectiveness.
2) Use the Taxonomy of Behaviour Change techniques for smoking cessation[13] to examine which behaviour change functions are most effective for smoking cessation in people with current depression.
3) Examine the difference in change in depression scores between intervention and control arms.

For peer review only

## METHODS

The study protocol has been registered in advance on the International Prospective Register of Systematic Reviews ((PROSPERO); ID: CRD42017070741; http://www.crd.york.ac.uk/PROSPERO/), and will be uploaded to bioRxiv (http://biorxiv.org/). All methods and study reporting will adhere to guidance described within the Cochrane Handbook for Systematic Reviews and Meta-analyses of Randomised Controlled Trials.[14]

### Search strategy

We will include relevant studies identified by a previously conducted Cochrane review of smoking cessation interventions for people with depression, and from the Cochrane review update due to commence this year.[9] Studies have been identified from Cochrane Central Register of Controlled trials (CENTRAL), MEDLINE, EMBASE, and PsycINFO using search terms related to 'depression', and 'tobacco' or 'smoking' as recommended by the Tobacco Addiction Group and the Cochrane Depression, Anxiety and Neurosis Group. See the Tobacco Addiction Group Module in The Cochrane Library for full search strategies and the list of other resources searched.[9] This search strategy will be updated for additional relevant studies published from 2013. RV is the lead author for the Cochrane review published in 2013, and will lead on the Cochrane update of this review. To avoid duplicating efforts across teams and given the high reliance of Cochrane methods, RV will share the eligible studies prior to data extraction of the Cochrane update. We predict that this will take place in early 2018.

### Inclusion criteria

Inclusion criteria are based on those outlined in the 2013 Cochrane review.[9]

- Study design: Randomised controlled trials only;
- Participants: Daily smokers with current depression, any definition of depression, no restrictions by physical or mental comorbidities;
- Intervention: Any smoking cessation intervention;
- Intervention delivery: Self-help, individual, group, internet;
- Control: Any (e.g., including self-help, no treatment, etc.);
- Outcome: Any ascertainment of smoking cessation;
- Follow-up: Follow up at a minimum of 6- months from the quit date.

### Outcomes

- Smoking status at final follow-up (same as the 2013 Cochrane review[9])
- Change in depression scores from baseline to final follow-up (not reported in the 2013 Cochrane review[9]).

For peer review only

**Data extraction**

We will use the following data as reported in the 2013 Cochrane review[9]:

- Trial methods - Study design, setting, country, randomisation methods.
- Participants - Number of participants per intervention group, definition of depression, type of smoker, comorbid conditions, age, sex, ethnicity, level of education, nicotine dependence, mean/median number of cigarettes per day (CPD), depression type and severity.
- Outcomes - Smoking cessation status, biochemical validation, depression scores, length of follow-up
- Measures of treatment effect, smoking cessation (Study aims 1 & 2): We will use the following outcome data as reported in the Cochrane review. The number of participants randomised to the intervention and control groups, and the number of participants who quit smoking in the intervention and control groups [14]

We will extract the following additional data not reported by in the 2013 Cochrane review[9]:

- Interventions - The number of and function of behaviour change techniques used (i.e. where sufficient details are not reported in text, we will attempt to obtain intervention protocols), presence or absence of TIDieR checklist items.
- Control - The number of and function of behaviour change techniques used (where sufficient details are not reported in text, we will attempt to obtain intervention protocols), presence or absence of TIDieR checklist items.
- Measures of treatment effect, depression symptoms (Study aim 3): For each trial arm, we will obtain mean depression scores and measure of variance at baseline and follow-up, mean differences and measures of variance from baseline to follow-up, or differences in change between trial arms' scores from baseline to follow-up and measures of variance.

**Coding of Template for Intervention Description and Replication checklist (TIDieR)**

The 2013 Cochrane review[9] did not extract any information relevant to the TIDieR checklist, these data are new to this review.

For study aim 1 we will use the Template for Intervention Description and Replication checklist (TIDieR)[12] to determine if variations in delivery of mood management components impact on intervention effectiveness. We will use a modified version of the template as not all questions on the checklist are useful in the context of this study (Table 1) (e.g. "Describe any rationale, theory, or goal of the elements essential to the intervention"). Coding will be conducted separately by two researchers to confirm agreement.

**Coding of behaviour change intervention functions using the Behaviour Change Taxonomy (BCT)**

The 2013 Cochrane review[9] did not extract any information relevant to the BCT, these data are new to this review.

We will categorise behaviour change techniques according to their function and record whether the function was either absent or present during intervention delivery[13] (Table 2). Coding will be conducted separately by two researchers to confirm agreement.

For peer review only

**Measures of treatment effect**

- Smoking cessation (Study aims 1 & 2): We will present treatment effects as risk ratios (RR) and 95% confidence intervals[14]. RRs will be calculated as follows: (number of participants who quit smoking in the intervention group/number of participants randomised to intervention group) divided by (number of participants who quit smoking in control group/number of participants randomised to the control group).
- Difference in change in depression scores between trial arms (Study aim 3): We will present the standardised mean difference (SMD), and 95% confidence intervals of change in depression scores between treatment arms, from baseline to follow-up.

**Analysis**

We will conduct analyses using Stata 14 or Revman software.

The following analytical procedures for each study aim are as follows:

1. Do variations in delivery of mood management components impact on intervention effectiveness: If there are sufficient data, we will conduct random effects meta-regression using the `metareg` command[15] in which modified TIDieR Checklist items (see Table 1) will be regressed on the study's effect estimate. First, univariate analyses will be conducted to determine the association between each item and the study effect size. Subsequently, items with the strongest association will be added to the meta-regression model first, and all other variables will be added in turn regardless of significance in the univariate model.
2. Use the Taxonomy of Behaviour Change techniques for smoking cessation to examine which behaviour change functions are most effective for smoking cessation in people with current depression: If there are sufficient data, we will conduct random effects meta-regression using the `metareg` command[15] in which behaviour change functions (see Table 2) will be regressed on the study's effect estimate. First, univariate analyses will be conducted to determine the association between each intervention component and the study effect size. Subsequently, variables with the strongest association will be added to the meta-regression model first, and all other variables will be added in turn regardless of significance in the univariate model.
3. Examine the difference in change in depression scores between intervention and control arms: If there are sufficient data, we will use a generic inverse variance random effects model to pool the standardised mean difference (SMD) of change in depression scores in treatment and control arms, from baseline to follow-up. We will use a random effects model as it incorporates heterogeneity both within and between studies.

Statistical heterogeneity: We will quantify statistical heterogeneity using $I^2$ which describes the percentage (%) of between-study variability due to heterogeneity rather than chance; values over 50% suggest substantial heterogeneity, and values over 75% suggest considerable heterogeneity.[14] $Tau^2$ will be used to test whether differences between studies' effect estimates are compatible with chance alone.[16]

Sensitivity and subgroup analyses: We will conduct sensitivity analyses to examine if the following study characteristics influence the meta-analysis results: study quality (as measured by Cochrane's Risk of Bias tool), loss-to-follow-up, and severity of depression.

Assessment of publication bias: We will examine funnel plots for evidence of asymmetry and conduct egger tests for evidence of small study bias using the `metabias` command.[15]

For peer review only

**Dissemination**

We will disseminate the findings of this work at international and national conferences, and to the UK Centre for Tobacco and Alcohol Studies Smokers' Panel.

For peer review only

## DISCUSSION

We will use the methods described in this protocol to determine: 1) if variations in delivery of mood management impact on smoking cessation intervention effectiveness in people with depression, 2) to examine which behaviour change functions are most effective for smoking cessation in people with depression, and 3) examine the difference in change in depression scores between intervention and control arms.

We hold no strong hypotheses about which variations in mood management delivery/behaviour change functions will impact on treatment effectiveness. Potentially, intervention functions that focus on improving motivation to quit may strengthen the association between intervention and smoking cessation, as poor motivation is a hallmark symptom of depression. We do predict that at minimum smoking cessation Intervention will not be associated with a worsening in depression, and that intervention may be associated with an improvement in depression scores when compared to control.[11]

### Clinical applications

If we are able to show that certain variations in delivery of mood management or behavioural support for smoking cessation are associated with higher abstinence rates, these data can be used by clinicians and researchers to optimise smoking cessation programmes for people with depression. Second, data pertaining to the impact of helping smokers with depression to quit smoking on depression symptoms will be imperative to smokers and clinicians.

For peer review only

For peer review only

**ETHICAL APPROVAL**

Ethical approval is not required for this study.

**ACKNOWLEDGMENTS**

None.

**COMPETING INTERESTS**

All authors completed ICMJE Form for Disclosure of Potential Conflicts of Interest. GT, DT, RV, DK, BS report no competing interests. PA reports non-financial support from GSK, outside the submitted work. MM reports grants from Pfizer, outside the submitted work.

**FUNDING**

GT is funded by Cancer Research UK Population Researcher Postdoctoral Fellowship award (reference: C56067/A21330). PA is supported by the NIHR Biomedical Research Centre and the NIHR CLAHRC. RV reports no funding for this work. BS and DK are funded by The University of Bristol. MRM would like to acknowledge funding from The MRC Integrative Epidemiology Unit at the University of Bristol which is supported by the Medical Research Council and the University of Bristol (MC_UU_12013/6). GT, MRM and PA are members of the UK Centre for Tobacco and Alcohol Studies, a UKCRC Public Health Research: Centre of Excellence. Funding from the British Heart Foundation, Cancer Research UK, Economic and Social Research Council, Medical Research Council, and the National Institute for Health Research, under the auspices of the UK Clinical Research Collaboration, is gratefully acknowledged. The funders have had no role in developing the protocol or study design.

**CONTRIBUTORSHIP STATEMENT**

All authors contributed to writing the manuscript and reviewed the final draft. GT, PA, RV, DK, BS, MM all contributed towards study design. DT contributed towards writing the manuscript. GT and MM act as the guarantors of this review.

## REFERENCES

1. Organization WH, Press WHO. *WHO Report on the Global Tobacco Epidemic: Warning about the Dangers of Tobacco*. Geneva, Switzerland; 2011.
2. Physicians RC of, Psychiatrists RC of, Physicians RC of. *Smoking and Mental Health*. London, UK; 2013.
3. Chang C, Hayes R, Perera G, et al. Life expectancy at birth for people with serious mental illness and other major disorders from a secondary mental health care case register in London. *PLoS One*. 2011;6(5):e19590. doi:doi:10.1371/journal.pone.0019590.
4. Lawrence D, Mitrou F, Zubrick SR, et al. Smoking and mental illness: results from population surveys in Australia and the United States. *BMC Public Health*. 2009;9(1):285. doi:10.1186/1471-2458-9-285.
5. Lasser K, Boyd J, Woolhandler S, Himmelstein D, McCormick D, Bor D. Smoking and mental illness: a population-based prevalence study. *JAMA*. 2000;284(20):2606-2610.
6. Johnson KA, Bonn-Miller MO, Leyro TM, Zvolensky MJ. Anxious Arousal and Anhedonic Depression Symptoms and the Frequency of Current Marijuana Use: Testing the Mediating Role of Marijuana-Use Coping Motives Among Active Users. *J Stud Alcohol Drugs*. 2009;70(4):543-550. wos:000267230100007.
7. Zhou X, Nonnemaker J, Sherrill B, Gilsenan AW, Coste F, West R. Attempts to quit smoking and relapse: factors associated with success or failure from the ATTEMPT cohort study. *Addict Behav*. 2009;34(4):365-373. http://ukpmc.ac.uk/abstract/MED/19097706.
8. Social Care LG and CPD. *Closing the Gap: Priorities for Essential Change in Mental Health* . London, UK: Department of Health; 2014.
9. van der Meer RM, Willemsen MC, Smit F, Cuijpers P. Smoking cessation interventions for smokers with current or past depression. *Cochrane Libr*. 2013.
10. Sheals K, Tombor I, McNeill A, Shahab L. A mixed-method systematic review and meta-analysis of mental health professionals' attitudes toward smoking and smoking cessation amongst people with mental illnesses. *Addiction*. 2016:n/a-n/a. doi:10.1111/add.13387.
11. Taylor G, McNeill A, Girling A, Farley A, Lindson N, Aveyard P. Change in mental health after smoking cessation: systematic review and meta-analysis. *BMJ*. 2014;348.
12. Hoffmann TC, Glasziou PP, Boutron I, et al. Better reporting of interventions: template for intervention description and replication (TIDieR) checklist and guide. *BMJ*. 2014;348:g1687. http://www.ncbi.nlm.nih.gov/pubmed/24609605. Accessed May 30, 2017.
13. Michie S, Hyder N, Walia A, West R. Development of a taxonomy of behaviour change techniques used in individual behavioural support for smoking cessation. *Addict Behav*. 2011;36:315-319.
14. Higgins J, Green S. *Cochrane Handbook for Systematic Reviews of Interventions Version 5.1.0*. The Cochrane Collaboration; 2011.
15. Sterne, Jonathan AC, Michael J. Bradburn and ME. Meta–Analysis in Stata™. In: *Systematic Reviews in Health Care: Meta-Analysis in Context*. Second Edi. ; 2008:347-369.
16. Harbord R, Higgins J. METAREG: Stata module to perform meta-analysis regression. *Stat Softw Components*. 2009. http://ideas.repec.org/c/boc/bocode/s446201.html%5Cnhttp://fmwww.bc.edu/repec/bocode/m/metareg.ado%5Cnhttps://ideas.repec.org/c/boc/bocode/s446201.html.

For peer review only

**TABLES**

**Table 1. Modified version of Template for Intervention Description and Replication checklist (TIDieR)[12] for use in meta-regression analysis***

| Item | Categories |
|---|---|
| Materials for mood management (i.e., physical or informational materials used) | Paper-based information, website, homework, diary, audio information, etc. |
| Procedures for mood management: activities, procedures or activities used in the intervention to support activities | Relaxation techniques, mood monitoring, etc. |
| Did the participant see the same intervention provider for all mood management sessions? | Yes, no |
| Mood management provider | Nurse, psychologist, GP, counsellor, etc. |
| Training given to intervention provider? | Yes, no |
| Level of education of intervention provider | BSc, MSc, PhD |
| Mode of mood management intervention delivery | Individual, group |
| Location of mood management intervention | Hospital, participant's home, GP surgery, university, etc. |
| Number of mood management sessions | Continuous variable |
| Length of mood management session (minutes) | Continuous variable |
| Was the mood management intervention tailored to participant? | Yes, no |
| Number of mood management sessions tailored to participant? | Continuous variable |
| Was participant adherence to mood management intervention measured? | Yes, no |
| Did participants to adhere to mood management intervention. | Yes, no, or % |
| Was therapist adherence to mood management intervention measured? (Y/N) | Yes, no |
| Did therapists adhere to mood management programme? (Y/N) | Yes, no, or % |

* The categories are likely to be further developed during data extraction to include new items.

For peer review only

| Table 2. Example of Behaviour change functions and techniques[13] | |
|---|---|
| **Behaviour change function** | **Examples of technique** |
| Specific focus on behaviour and addressing motivation | Provide information on consequences of smoking and smoking cessation |
| | Boost motivation and self-efficacy |
| | Provide feedback on current behaviour |
| Specific focus on behaviour and maximising self-regulatory capacity/skills | Advise on changing routine |
| | Advise on environmental restructuring |
| | Set graded tasks |

342

For peer review only

**PRISMA-P (Preferred Reporting Items for Systematic review and Meta-Analysis Protocols) 2015 checklist: recommended items to address in a systematic review protocol***

| Section and topic | Item No | Checklist item | Page |
|---|---|---|---|
| **ADMINISTRATIVE INFORMATION** | | | |
| Title: | | | |
| Identification | 1a | Identify the report as a protocol of a systematic review | 1 |
| Update | 1b | If the protocol is for an update of a previous systematic review, identify as such | n/a |
| Registration | 2 | If registered, provide the name of the registry (such as PROSPERO) and registration number | 2, 5 |
| Authors: | | | |
| Contact | 3a | Provide name, institutional affiliation, e-mail address of all protocol authors; provide physical mailing address of corresponding author | 1 |
| Contributions | 3b | Describe contributions of protocol authors and identify the guarantor of the review | 8 |
| Amendments | 4 | If the protocol represents an amendment of a previously completed or published protocol, identify as such and list changes; otherwise, state plan for documenting important protocol amendments | n/a |
| Support: | | | |
| Sources | 5a | Indicate sources of financial or other support for the review | 8 |
| Sponsor | 5b | Provide name for the review funder and/or sponsor | 8 |
| Role of sponsor or funder | 5c | Describe roles of funder(s), sponsor(s), and/or institution(s), if any, in developing the protocol | 8 |
| **INTRODUCTION** | | | |
| Rationale | 6 | Describe the rationale for the review in the context of what is already known | 4 |
| Objectives | 7 | Provide an explicit statement of the question(s) the review will address with reference to participants, interventions, comparators, and outcomes (PICO) | 4 |
| **METHODS** | | | |
| Eligibility criteria | 8 | Specify the study characteristics (such as PICO, study design, setting, time frame) and report characteristics (such as years considered, language, publication status) to be used as criteria for eligibility for the review | 5-6 |
| Information sources | 9 | Describe all intended information sources (such as electronic databases, contact with study authors, trial registers or other grey literature sources) with planned dates of coverage | 5 |
| Search strategy | 10 | Present draft of search strategy to be used for at least one electronic database, including planned limits, such that it could be repeated | n/a |

For peer review only

| | | | |
|---|---|---|---|
| Study records: | | | |
| Data management | 11a | Describe the mechanism(s) that will be used to manage records and data throughout the review | 6 |
| Selection process | 11b | State the process that will be used for selecting studies (such as two independent reviewers) through each phase of the review (that is, screening, eligibility and inclusion in meta-analysis) | n/a |
| Data collection process | 11c | Describe planned method of extracting data from reports (such as piloting forms, done independently, in duplicate), any processes for obtaining and confirming data from investigators | 6 |
| Data items | 12 | List and define all variables for which data will be sought (such as PICO items, funding sources), any pre-planned data assumptions and simplifications | 6 |
| Outcomes and prioritization | 13 | List and define all outcomes for which data will be sought, including prioritization of main and additional outcomes, with rationale | 5-6 |
| Risk of bias in individual studies | 14 | Describe anticipated methods for assessing risk of bias of individual studies, including whether this will be done at the outcome or study level, or both; state how this information will be used in data synthesis | 7 |
| Data synthesis | 15a | Describe criteria under which study data will be quantitatively synthesised | 6-7 |
| | 15b | If data are appropriate for quantitative synthesis, describe planned summary measures, methods of handling data and methods of combining data from studies, including any planned exploration of consistency (such as $I^2$, Kendall's τ) | 6-7 |
| | 15c | Describe any proposed additional analyses (such as sensitivity or subgroup analyses, meta-regression) | 7 |
| | 15d | If quantitative synthesis is not appropriate, describe the type of summary planned | 6-7 |
| Meta-bias(es) | 16 | Specify any planned assessment of meta-bias(es) (such as publication bias across studies, selective reporting within studies) | 6-7 |
| Confidence in cumulative evidence | 17 | Describe how the strength of the body of evidence will be assessed (such as GRADE) | n/a |

*** It is strongly recommended that this checklist be read in conjunction with the PRISMA-P Explanation and Elaboration (cite when available) for important clarification on the items. Amendments to a review protocol should be tracked and dated. The copyright for PRISMA-P (including checklist) is held by the PRISMA-P Group and is distributed under a Creative Commons Attribution Licence 4.0.**

*From: Shamseer L, Moher D, Clarke M, Ghersi D, Liberati A, Petticrew M, Shekelle P, Stewart L, PRISMA-P Group. Preferred reporting items for systematic review and meta-analysis protocols (PRISMA-P) 2015: elaboration and explanation. BMJ. 2015 Jan 2;349(jan02 1):g7647.*

For peer review only

BMJ Open

# Impact of variation in intervention delivery and intervention functions on the effectiveness of behavioural and mood management interventions for smoking cessation in people with depression: A systematic review and meta-analysis protocol.

For peer review only

| | |
|---|---|
| Journal: | *BMJ Open* |
| Manuscript ID | bmjopen-2017-018617.R2 |
| Article Type: | Protocol |
| Date Submitted by the Author: | 26-Sep-2017 |
| Complete List of Authors: | Taylor, Gemma; University of Bristol, Medical Research Council Integrative Epidemiology Unit, UK Centre for Tobacco and Alcohol Studies, School of Experimental Psychology<br>Aveyard, Paul; University of Oxford, UK Centre for Tobacco and Alcohol Studies, Primary Care Health Sciences<br>Van der Meer, Regina ; Public Health Service of Haaglanden (GGD Haaglanden), Epidemiology; Maastricht University, CAPHRI<br>Toze, Daniel; University of Bristol, Medical Research Council Integrative Epidemiology Unit, UK Centre for Tobacco and Alcohol Studies, School of Experimental Psychology<br>Stuijfzand, Bobby ; University of Bristol, Jean Golding Institute for Data-Intensive Research<br>Kessler, David; University of Bristol, Centre for Academic Mental Health<br>Munafo, Marcus; University of Bristol, Medical Research Council Integrative Epidemiology Unit, UK Centre for Tobacco and Alcohol Studies, School of Experimental Psychology |
| <b>Primary Subject Heading</b>: | Smoking and tobacco |
| Secondary Subject Heading: | Mental health |
| Keywords: | Tobacco, Smoking cessation, Depression, Systematic review, Protocol, Intervention |
| | |
| Note: The following files were submitted by the author for peer review, but cannot be converted to PDF. You must view these files (e.g. movies) online. | |
| Electronic search.odt | |

SCHOLARONE™
Manuscripts

**TITLE**

# Impact of variation in intervention delivery and intervention functions on the effectiveness of behavioural and mood management interventions for smoking cessation in people with depression: A systematic review and meta-analysis protocol.


**AUTHORS**

Gemma Taylor, Research Fellow [1,2]; Paul Aveyard, Professor of Behavioural Medicine [3]; Regina Van der Meer, Epidemiologist, Health Scientist [4,5]; Daniel Toze, Research Assistant [1,2]; Bobby Stuijfzand, Data Science Specialist [6]; David Kessler, Reader in Primary Care [7]; Marcus Munafò, Professor of Biological Psychology [1,2]

**AFFILIATIONS**

[1] Medical Research Council Integrative Epidemiology Unit at the University of Bristol, Bristol, BS8 2BN, United Kingdom.

[2] UK Centre for Tobacco and Alcohol Studies, School of Experimental Psychology, University of Bristol, 12a Priory Road, Bristol, BS8 1TU, United Kingdom.

[3] UK Centre for Tobacco and Alcohol Studies, Nuffield Department of Primary Care Health Sciences, University of Oxford, Oxford, OX1 2ET, United Kingdom.

[4] Public Health Service of Haaglanden (GGD Haaglanden), Epidemiology, The Hague, Netherlands.

[5] Maastricht University, CAPHRI, Maastricht, Netherlands

[6] Jean Golding Institute for Data-Intensive Research, University of Bristol, Royal Fort House, Bristol, BS8 1UH, United Kingdom.

[7] Centre for Academic Mental Health, University of Bristol, Oakfield House, Oakfield Grove, Bristol, BS8 2BN, United Kingdom.

**CORRESPONDING AUTHOR**

Gemma Taylor, gemma.taylor@bristol.ac.uk, MRC Integrative Epidemiology Unit, UK Centre for Tobacco and Alcohol Studies, School of Experimental Psychology, University of Bristol, 12a Priory Road, Bristol, BS8 1TU, United Kingdom.



**WORD COUNT**

1379

**KEYWORDS**

Tobacco, Smoking cessation, Depression, Systematic review, Protocol, Intervention


For peer review only

## ABSTRACT

### Introduction

Tobacco is the world's leading preventable cause of disease and death. People with depression are twice as likely to smoke and are less responsive to standard tobacco treatments as compared to the general population. A Cochrane systematic review of randomised controlled trials of smoking cessation treatment for smokers with current or historical depression found that adding mood management to usual smoking treatment improved quit rates. However, the review did not examine if variation in intervention delivery or intervention functions impacted on treatment effectiveness.

With the aim of providing information to develop tailored approaches to treating smoking for people with current depression we will add-on to the Cochrane review in three ways: 1) Use the Template for Intervention Description and Replication checklist to determine if variations in mood management delivery impact on intervention effectiveness, 2) Use the Taxonomy of Behaviour Change Techniques for smoking cessation to examine which behaviour change functions are most effective for smoking cessation in people with current depression, 3) Examine the difference in change in depression scores between intervention and control arms.

### Methods and Analysis

We will include randomised controlled trials of smokers with current depression as identified by the previous Cochrane review and the in-progress update of the Cochrane review. We will use meta-regression to examine 1) if variations in delivery of mood management impact on smoking cessation intervention effectiveness, 2) determine which behaviour change functions are most effective for smoking cessation and 3) use meta-analysis of the difference in change in depression scores between treatment arms from baseline to follow-up to determine if offering smoking cessation treatment causes psychological harm.

### Ethics and Dissemination

Ethical approval is not required for this study. We will disseminate the findings of this work at conferences, and to relevant patient panels.

### Registration details

PROSPERO ID: CRD42017070741

For peer review only

**STRENGTHS AND LIMITATIONS OF THIS STUDY**

- We will examine the impact of variation in intervention delivery and intervention functions on treatment effectiveness using peer-reviewed checklists: The Behaviour Change Taxonomy, and Template for Intervention Description and Replication.
- The study design may suffer from low power and/or publication bias.

For peer review only

## BACKGROUND

Tobacco is the world's leading preventable cause of disease and death.[1] In the UK and in other developed nations smoking prevalence has declined substantially in the general population, but has remained largely unchanged in those with mental health problems resulting in an excess burden of smoking-related mortality in this group.[2,3] People with depression are twice as likely to smoke[4,5] and are less responsive to standard tobacco treatments than are the general population[6,7] leading to urgent calls for targeted smoking interventions.[8]

The Cochrane Group conducted a systematic review and meta-analysis of smoking cessation interventions for smokers with past or present depression. The review included pharmacological and behavioural interventions to aid cessation and found that adding mood management to a usual smoking treatment (e.g., nicotine replacement therapy, telephone counselling, self-help website) moderately increased smoking cessation rates in people with current depression compared to usual smoking treatment alone, reporting a risk ratio of 1.47 (95% confidence interval: 1.13 to 1.92).[9] The review highlighted the importance of adding psychological techniques to handle depressive symptoms in standard smoking treatments for people with depression. However, in the meta-analysis there was variation between the included studies' direction of effect and it is possible that this variation may be in part related to differences in intervention delivery or intervention functions, for example. Further investigation into these potential modifiers will provide useful information for development of smoking cessation interventions for people with current depression.[9]

In addition, the review did not examine the impact of behavioural or psychological smoking cessation interventions on depression symptoms. This is an important question as many clinicians believe that smoking may offer mental health benefits, or that their patients' mental health may deteriorate upon cessation.[10] However, there are data from meta-analyses of cohort studies indicating that quitting smoking may improve depression,[11] but due to common pitfalls of observational data one cannot be sure that this is a causal association. If treating smoking is found to not worsen depression, then these data can be used to assure clinicians that they are not causing psychological harm by helping their patients to quit smoking.

In our review, we aim to add-on to the 2013 Cochrane review[9] in three ways. We will:

1) Use the Template for Intervention Description and Replication checklist (TIDieR)[12] to determine if variations in mood management delivery impact on intervention effectiveness in people with depression.
2) Use the Taxonomy of Behaviour Change techniques for smoking cessation[13] to examine which behaviour change functions are most effective for smoking cessation in people with current depression.
3) Examine the difference in change in depression scores between intervention and control arms in people with current depression.

For peer review only

## METHODS

The study protocol has been registered in advance on the International Prospective Register of Systematic Reviews ((PROSPERO); ID: CRD42017070741; http://www.crd.york.ac.uk/PROSPERO/). All methods and study reporting will adhere to guidance described within the Cochrane Handbook for Systematic Reviews and Meta-analyses of Randomised Controlled Trials.[14]

### Search strategy

We will include relevant studies identified by a previously conducted Cochrane review of smoking cessation interventions for people with depression, and from the Cochrane review update due to commence this year.[9] Studies have been identified from the Cochrane Central Register of Controlled trials (CENTRAL), MEDLINE, EMBASE, and PsycINFO using search terms related to 'depression', and 'tobacco' or 'smoking' as recommended by the Tobacco Addiction Group and the Cochrane Depression, Anxiety and Neurosis Group. See the Tobacco Addiction Group Module in The Cochrane Library for full search strategies and the list of other resources searched.[9] This search strategy will be updated for additional relevant studies published from 2013. RV is the lead author for the Cochrane review published in 2013, and will lead on the Cochrane update of this review. To avoid duplicating efforts across teams and given the high reliance of Cochrane methods, RV will share the eligible studies prior to data extraction of the Cochrane update. We predict that this will take place in early 2018.

### Inclusion criteria

Inclusion criteria are based on those outlined in the 2013 Cochrane review.[9]

- Study design: Randomised controlled trials only;
- Participants: Daily smokers with current depression, any definition of depression, no restrictions by physical or mental comorbidities;
- Intervention: Any smoking cessation intervention;
- Intervention delivery: Self-help, individual, group, internet;
- Control: Any (e.g., including self-help, no treatment, etc.);
- Outcome: Any ascertainment of smoking cessation;
- Follow-up: Follow up at a minimum of 6- months from the quit date.

### Outcomes

- Smoking status at final follow-up (same as the 2013 Cochrane review[9])
- Change in depression scores from baseline to final follow-up (not reported in the 2013 Cochrane review[9]).

For peer review only

**Data extraction**

We will use the following data as reported in the 2013 Cochrane review[9]:

- Trial methods - Study design, setting, country, randomisation methods.
- Participants - Number of participants per intervention group, definition of depression, type of smoker, comorbid conditions, age, sex, ethnicity, level of education, nicotine dependence, mean/median number of cigarettes per day (CPD), depression type and severity.
- Outcomes - Smoking cessation status, biochemical validation, depression scores, length of follow-up.
- Measures of treatment effect, smoking cessation (Study aims 1 & 2): We will use the following outcome data as reported in the Cochrane review. The number of participants randomised to the intervention and control groups, and the number of participants who quit smoking in the intervention and control groups [14]

We will extract the following additional data not reported by in the 2013 Cochrane review[9]:

- Interventions - The number of and function of behaviour change techniques used (i.e. where sufficient details are not reported in text, we will attempt to obtain intervention protocols), and presence or absence of TIDieR checklist items.
- Control - The number of and function of behaviour change techniques used (i.e. where sufficient details are not reported in text, we will attempt to obtain intervention protocols), and presence or absence of TIDieR checklist items.
- Measures of treatment effect, depression symptoms (Study aim 3): For each trial arm, we will obtain mean depression scores and measure of variance at baseline and follow-up, mean differences and measures of variance from baseline to follow-up, or differences in change between trial arms' scores from baseline to follow-up and measures of variance.

**Coding of Template for Intervention Description and Replication checklist (TIDieR)**

The 2013 Cochrane review[9] did not extract any information relevant to the TIDieR checklist[12], these data are new to this review.

For study aim 1 we will use the TIDieR checklist[12] to determine if variations in mood management delivery impact on intervention effectiveness. We will use a modified version of TIDieR as not all items on the checklist are useful in the context of this study (Table 1) (e.g. "Describe any rationale, theory, or goal of the elements essential to the intervention"). Coding will be conducted separately by two researchers to confirm agreement.

**Coding of behaviour change intervention functions using the Behaviour Change Taxonomy (BCT)**

The 2013 Cochrane review[9] did not extract any information relevant to the BCT[13], these data are new to this review.

For study aim 2 we will code the number of behaviour change techniques, categorise the behaviour change techniques according to their function, and record whether the function was either absent or present during intervention delivery[13] (Table 2). Coding will be conducted separately by two researchers to confirm agreement.

For peer review only

### Measures of treatment effect

- Smoking cessation (Study aims 1 & 2): We will present treatment effects as risk ratios (RR) and 95% confidence intervals[14]. RRs will be calculated as follows: (number of participants who quit smoking in the intervention group/number of participants randomised to intervention group) divided by (number of participants who quit smoking in control group/number of participants randomised to the control group).
- Difference in change in depression scores between trial arms (Study aim 3): We will present the standardised mean difference (SMD), and 95% confidence intervals of change in depression scores between treatment arms, from baseline to follow-up.

### Analysis

We will conduct analyses using Stata 14 or Revman software, and use the following analytical procedures to address each study aim:

1. Do variations mood management delivery impact on intervention effectiveness: If there are sufficient data, we will conduct random effects meta-regression models using the `metareg` command[15] in which modified TIDieR checklist items (see Table 1) will be regressed on the study's effect estimate. First, univariate analyses will be conducted to determine the association between each item and the study effect size. Subsequently, items with the strongest association will be added to the meta-regression model first, and all other variables will be added in turn regardless of significance in the univariate model.
2. Use the Taxonomy of Behaviour Change techniques for smoking cessation to examine which behaviour change functions are most effective for smoking cessation in people with current depression: If there are sufficient data, we will conduct random effects meta-regression models using the `metareg` command[15] in which behaviour change functions (see Table 2) will be regressed on the study's effect estimate. First, univariate analyses will be conducted to determine the association between each intervention function and the study effect size. Subsequently, variables with the strongest association will be added to the meta-regression model first, and all other variables will be added in turn regardless of significance in the univariate model.
3. Examine the difference in change in depression scores between intervention and control arms: If there are sufficient data, we will use a generic inverse variance random effects model to pool the standardised mean difference (SMD) of change in depression scores in treatment and control arms, from baseline to follow-up. We will use a random effects model as it incorporates heterogeneity both within and between studies.

Statistical heterogeneity: We will quantify statistical heterogeneity using $I^2$ which describes the percentage (%) of between-study variability due to heterogeneity rather than chance; values over 50% suggest substantial heterogeneity, and values over 75% suggest considerable heterogeneity.[14] $Tau^2$ will be used to test whether differences between studies' effect estimates are compatible with chance alone.[16]

Sensitivity and subgroup analyses: We will conduct sensitivity analyses to examine if the following study characteristics influence the meta-analysis results: study quality (as measured by Cochrane's Risk of Bias tool), loss-to-follow-up, and severity of depression.

Assessment of publication bias: We will examine funnel plots for evidence of asymmetry and conduct egger tests for evidence of small study bias using the `metabias` command.[15]

For peer review only

**Ethics and dissemination**

We will not require ethical approval for the conduct of this systematic review and meta-analysis. We will disseminate the findings of this work at international and national conferences, and to the UK Centre for Tobacco and Alcohol Studies Smokers' Panel.

For peer review only

## DISCUSSION

We will use the methods described in this protocol to determine: 1) if variations in delivery of mood management impact on smoking cessation intervention effectiveness in people with depression, 2) to examine which behaviour change functions are most effective for smoking cessation in people with depression, and 3) examine the difference in change in depression scores between intervention and control arms.

We hold no strong hypotheses about which variations in mood management delivery/behaviour change functions will impact on treatment effectiveness. Potentially, intervention functions that focus on improving motivation to quit may strengthen the association between intervention and smoking cessation, as poor motivation is a hallmark symptom of depression. We do predict that at minimum smoking cessation intervention will not be associated with a worsening in depression, and that intervention may be associated with an improvement in depression scores when compared to control.[11]

### Clinical applications

If we can show that certain variations in delivery of mood management or behavioural support for smoking cessation are associated with higher abstinence rates, these data can be used by clinicians and researchers to optimise smoking cessation programmes for people with depression. Second, data pertaining to the impact of helping smokers with depression to quit smoking on depression symptoms will be imperative to smokers and clinicians.

For peer review only

**ETHICAL APPROVAL**

Ethical approval is not required for this study.

**ACKNOWLEDGMENTS**

None.

**COMPETING INTERESTS**

All authors completed ICMJE Form for Disclosure of Potential Conflicts of Interest. GT, DT, RV, DK, BS report no competing interests. PA reports non-financial support from GSK, outside the submitted work. MM reports grants from Pfizer, outside the submitted work.

**FUNDING**

GT is funded by Cancer Research UK Population Researcher Postdoctoral Fellowship award (reference: C56067/A21330). PA is supported by the NIHR Biomedical Research Centre and the NIHR CLAHRC. RV reports no funding for this work. BS and DK are funded by The University of Bristol. MRM would like to acknowledge funding from The MRC Integrative Epidemiology Unit at the University of Bristol which is supported by the Medical Research Council and the University of Bristol (MC_UU_12013/6). GT, MRM and PA are members of the UK Centre for Tobacco and Alcohol Studies, a UKCRC Public Health Research: Centre of Excellence. Funding from the British Heart Foundation, Cancer Research UK, Economic and Social Research Council, Medical Research Council, and the National Institute for Health Research, under the auspices of the UK Clinical Research Collaboration, is gratefully acknowledged. The funders have had no role in developing the protocol or study design.

**CONTRIBUTORSHIP STATEMENT**

All authors contributed to writing the manuscript and reviewed the final draft. GT, PA, RV, DK, BS, MM all contributed towards study design. DT contributed towards writing the manuscript. GT and MM act as the guarantors of this review.

For peer review only

## REFERENCES


1. Organization WH, Press WHO. *WHO Report on the Global Tobacco Epidemic: Warning about the Dangers of Tobacco*. Geneva, Switzerland; 2011.
2. Physicians RC of, Psychiatrists RC of, Physicians RC of. *Smoking and Mental Health*. London, UK; 2013.
3. Chang C, Hayes R, Perera G, et al. Life expectancy at birth for people with serious mental illness and other major disorders from a secondary mental health care case register in London. *PLoS One*. 2011;6(5):e19590. doi:doi:10.1371/journal.pone.0019590.
4. Lawrence D, Mitrou F, Zubrick SR, et al. Smoking and mental illness: results from population surveys in Australia and the United States. *BMC Public Health*. 2009;9(1):285. doi:10.1186/1471-2458-9-285.
5. Lasser K, Boyd J, Woolhandler S, Himmelstein D, McCormick D, Bor D. Smoking and mental illness: a population-based prevalence study. *JAMA*. 2000;284(20):2606-2610.
6. Johnson KA, Bonn-Miller MO, Leyro TM, Zvolensky MJ. Anxious Arousal and Anhedonic Depression Symptoms and the Frequency of Current Marijuana Use: Testing the Mediating Role of Marijuana-Use Coping Motives Among Active Users. *J Stud Alcohol Drugs*. 2009;70(4):543-550. wos:000267230100007.
7. Zhou X, Nonnemaker J, Sherrill B, Gilsenan AW, Coste F, West R. Attempts to quit smoking and relapse: factors associated with success or failure from the ATTEMPT cohort study. *Addict Behav*. 2009;34(4):365-373. http://ukpmc.ac.uk/abstract/MED/19097706.
8. Social Care LG and CPD. *Closing the Gap: Priorities for Essential Change in Mental Health* . London, UK: Department of Health; 2014.
9. van der Meer RM, Willemsen MC, Smit F, Cuijpers P. Smoking cessation interventions for smokers with current or past depression. *Cochrane Libr*. 2013.
10. Sheals K, Tombor I, McNeill A, Shahab L. A mixed-method systematic review and meta-analysis of mental health professionals' attitudes toward smoking and smoking cessation amongst people with mental illnesses. *Addiction*. 2016:n/a-n/a. doi:10.1111/add.13387.
11. Taylor G, McNeill A, Girling A, Farley A, Lindson N, Aveyard P. Change in mental health after smoking cessation: systematic review and meta-analysis. *BMJ*. 2014;348.
12. Hoffmann TC, Glasziou PP, Boutron I, et al. Better reporting of interventions: template for intervention description and replication (TIDieR) checklist and guide. *BMJ*. 2014;348:g1687. http://www.ncbi.nlm.nih.gov/pubmed/24609605. Accessed May 30, 2017.
13. Michie S, Hyder N, Walia A, West R. Development of a taxonomy of behaviour change techniques used in individual behavioural support for smoking cessation. *Addict Behav*. 2011;36:315-319.
14. Higgins J, Green S. *Cochrane Handbook for Systematic Reviews of Interventions Version 5.1.0*. The Cochrane Collaboration; 2011.
15. Sterne, Jonathan AC, Michael J. Bradburn and ME. Meta–Analysis in Stata™. In: *Systematic Reviews in Health Care: Meta-Analysis in Context*. Second Edi. ; 2008:347-369.
16. Harbord R, Higgins J. METAREG: Stata module to perform meta-analysis regression. *Stat Softw Components*. 2009. http://ideas.repec.org/c/boc/bocode/s446201.html%5Cnhttp://fmwww.bc.edu/repec/bocode/m/metareg.ado%5Cnhttps://ideas.repec.org/c/boc/bocode/s446201.html.


For peer review only

334 **TABLES**

335

| Table 1. Modified version of Template for Intervention Description and Replication checklist (TIDieR)[12] for use in meta-regression analysis* | |
|---|---|
| **Item** | **Categories** |
| Materials for mood management (i.e., physical or informational materials used) | Paper-based information, website, homework, diary, audio information, etc. |
| Procedures for mood management: activities, procedures or activities used in the intervention to support activities | Relaxation techniques, mood monitoring, etc. |
| Did the participant see the same intervention provider for all mood management sessions? | Yes, no |
| Mood management provider | Nurse, psychologist, GP, counsellor, etc. |
| Training given to intervention provider? | Yes, no |
| Level of education of intervention provider | BSc, MSc, PhD |
| Mode of mood management intervention delivery | Individual, group |
| Location of mood management intervention | Hospital, participant's home, GP surgery, university, etc. |
| Number of mood management sessions | Continuous variable |
| Length of mood management session (minutes) | Continuous variable |
| Was the mood management intervention tailored to participant? | Yes, no |
| Number of mood management sessions tailored to participant? | Continuous variable |
| Was participant adherence to mood management intervention measured? | Yes, no |
| Did participants to adhere to mood management intervention. | Yes, no, or % |
| Was therapist adherence to mood management intervention measured? (Y/N) | Yes, no |
| Did therapists adhere to mood management programme? (Y/N) | Yes, no, or % |
| * The categories are likely to be further developed during data extraction to include new items. | |

336

337

For peer review only

| Table 2. Example of Behaviour change functions and techniques[13] | |
|---|---|
| **Behaviour change function** | **Examples of technique** |
| Specific focus on behaviour and addressing motivation | Provide information on consequences of smoking and smoking cessation |
| | Boost motivation and self-efficacy |
| | Provide feedback on current behaviour |
| Specific focus on behaviour and maximising self-regulatory capacity/skills | Advise on changing routine |
| | Advise on environmental restructuring |
| | Set graded tasks |

For peer review only

**PRISMA-P (Preferred Reporting Items for Systematic review and Meta-Analysis Protocols) 2015 checklist: recommended items to address in a systematic review protocol***

| Section and topic | Item No | Checklist item | Page |
|---|---|---|---|
| **ADMINISTRATIVE INFORMATION** | | | |
| Title: | | | |
| Identification | 1a | Identify the report as a protocol of a systematic review | 1 |
| Update | 1b | If the protocol is for an update of a previous systematic review, identify as such | n/a |
| Registration | 2 | If registered, provide the name of the registry (such as PROSPERO) and registration number | 2, 5 |
| Authors: | | | |
| Contact | 3a | Provide name, institutional affiliation, e-mail address of all protocol authors; provide physical mailing address of corresponding author | 1 |
| Contributions | 3b | Describe contributions of protocol authors and identify the guarantor of the review | 8 |
| Amendments | 4 | If the protocol represents an amendment of a previously completed or published protocol, identify as such and list changes; otherwise, state plan for documenting important protocol amendments | n/a |
| Support: | | | |
| Sources | 5a | Indicate sources of financial or other support for the review | 8 |
| Sponsor | 5b | Provide name for the review funder and/or sponsor | 8 |
| Role of sponsor or funder | 5c | Describe roles of funder(s), sponsor(s), and/or institution(s), if any, in developing the protocol | 8 |
| **INTRODUCTION** | | | |
| Rationale | 6 | Describe the rationale for the review in the context of what is already known | 4 |
| Objectives | 7 | Provide an explicit statement of the question(s) the review will address with reference to participants, interventions, comparators, and outcomes (PICO) | 4 |
| **METHODS** | | | |
| Eligibility criteria | 8 | Specify the study characteristics (such as PICO, study design, setting, time frame) and report characteristics (such as years considered, language, publication status) to be used as criteria for eligibility for the review | 5-6 |
| Information sources | 9 | Describe all intended information sources (such as electronic databases, contact with study authors, trial registers or other grey literature sources) with planned dates of coverage | 5 |
| Search strategy | 10 | Present draft of search strategy to be used for at least one electronic database, including planned limits, such that it could be repeated | n/a |

For peer review only

| | | | |
|---|---|---|---|
| Study records: | | | |
| Data management | 11a | Describe the mechanism(s) that will be used to manage records and data throughout the review | 6 |
| Selection process | 11b | State the process that will be used for selecting studies (such as two independent reviewers) through each phase of the review (that is, screening, eligibility and inclusion in meta-analysis) | n/a |
| Data collection process | 11c | Describe planned method of extracting data from reports (such as piloting forms, done independently, in duplicate), any processes for obtaining and confirming data from investigators | 6 |
| Data items | 12 | List and define all variables for which data will be sought (such as PICO items, funding sources), any pre-planned data assumptions and simplifications | 6 |
| Outcomes and prioritization | 13 | List and define all outcomes for which data will be sought, including prioritization of main and additional outcomes, with rationale | 5-6 |
| Risk of bias in individual studies | 14 | Describe anticipated methods for assessing risk of bias of individual studies, including whether this will be done at the outcome or study level, or both; state how this information will be used in data synthesis | 7 |
| Data synthesis | 15a | Describe criteria under which study data will be quantitatively synthesised | 6-7 |
| | 15b | If data are appropriate for quantitative synthesis, describe planned summary measures, methods of handling data and methods of combining data from studies, including any planned exploration of consistency (such as $I^2$, Kendall's $\tau$) | 6-7 |
| | 15c | Describe any proposed additional analyses (such as sensitivity or subgroup analyses, meta-regression) | 7 |
| | 15d | If quantitative synthesis is not appropriate, describe the type of summary planned | 6-7 |
| Meta-bias(es) | 16 | Specify any planned assessment of meta-bias(es) (such as publication bias across studies, selective reporting within studies) | 6-7 |
| Confidence in cumulative evidence | 17 | Describe how the strength of the body of evidence will be assessed (such as GRADE) | n/a |

*** It is strongly recommended that this checklist be read in conjunction with the PRISMA-P Explanation and Elaboration (cite when available) for important clarification on the items. Amendments to a review protocol should be tracked and dated. The copyright for PRISMA-P (including checklist) is held by the PRISMA-P Group and is distributed under a Creative Commons Attribution Licence 4.0.**

*From: Shamseer L, Moher D, Clarke M, Ghersi D, Liberati A, Petticrew M, Shekelle P, Stewart L, PRISMA-P Group. Preferred reporting items for systematic review and meta-analysis protocols (PRISMA-P) 2015: elaboration and explanation. BMJ. 2015 Jan 2;349(jan02 1):g7647.*

For peer review only
